# Serotype b of *Aggregatibacter actinomycetemcomitans* triggers pro-inflammatory responses and amyloid beta secretion in hippocampal cells: a novel link between periodontitis and Alzheimer´s disease?

**DOI:** 10.1080/20002297.2019.1586423

**Published:** 2019-04-15

**Authors:** J. Díaz-Zúñiga, Y. Muñoz, S. Melgar-Rodríguez, J. More, B. Bruna, P. Lobos, G. Monasterio, R. Vernal, A. Paula-Lima

**Affiliations:** a Department of Conservative Dentistry, Faculty of Dentistry, Universidad de Chile, Santiago, Chile; b Biomedical Neuroscience Institute, Faculty of Medicine, Universidad de Chile, Santiago, Chile; c Aging Cellular Laboratory, Faculty of Sciences, Universidad de Chile, Santiago, Chile; d Dentistry Unit, Faculty of Health Sciences, Universidad Autónoma de Chile, Temuco, Chile; e Institute for Research in Dental Sciences, Faculty of Dentistry, Universidad de Chile, Santiago, Chile

**Keywords:** Periodontitis, Alzheimer´s disease, inflammation, cytokines, microglia, neurons

## Abstract

**Introduction:** Previous reports have proposed that Periodontal disease (PDis) predisposes to Alzheimer’s disease (AD), both highly prevalent pathologies among the elderly. The bacteria *Aggregatibacter actinomycetemcomitans* (*Aa*), associated with the most aggressive forms of PDis, are classified in different serotypes with distinct virulence according to the antigenicity of their lipopolysaccharide (LPS). **Methods: **Here, we determined the effects of purified LPS, from serotypes a, b or c of *Aa*, on primary cultures of microglia or mixed hippocampal cells. **Results: **We found that both culture types exhibited higher levels of inflammatory cytokines (IL-1β, IL-6 and TNFα) when treated with serotype b-LPS, compared with controls, as quantified by qPCR and/or ELISA. Also, cultures treated with serotype a-LPS displayed increased mRNA levels of the modulatory cytokines IL-4 and IL-10. Mixed hippocampal cultures treated with serotype b-LPS exhibited severe neuronal morphological changes and displayed increased levels of secreted Aβ_1-42_ peptide. These results indicate that LPS from different *Aa* serotypes triggers discriminatory immune responses, which differentially affect primary hippocampal cells. **Conclusion:** Altogether, our results show that treatment with serotype b-LPS triggers the secretion of proinflammatory cytokines by microglia, induces neurite shrinking, and increases the extracellular Aβ1-42 levels, all features strongly associated with the etiology of AD.

## Introduction

Periodontal disease (PDis) is a common chronic multi-bacterial infection affecting the supporting structures of the teeth, which results in a significant bacterial and inflammatory load in the body [1]. There is increasing clinical evidence that chronic PDis is closely related to the onset and progression of AD. The Third National Health and Nutrition Examination Survey (NHANES-III) indicated a cross-sectional association between a serologic marker of a common periodontitis pathogen, *Porphyromonas gingivalis (P. gingivalis*), and poor cognitive test performance among patients older than 60 years [2]. Also, the serum antibody levels to *P. gingivalis* are increased in participants diagnosed with AD in comparison with those in non-AD controls [3]. Furthermore, the presence of the major three periodontal bacteria (‘The Red complex’), including *Treponema denticola, Tannerella forsythia*, and *P. gingivalis* was detected in the brain tissue of individuals with and without dementia [4], but only the levels of LPS from *P. gingivalis* were significantly higher in the brain tissue from AD cases, compared to non-AD control tissues. More recent reports have shown that oral infection with *P. gingivalis* exacerbates pathological features in AD transgenic mice [5] and that this bacterium induces memory impairment and age-dependent neuroinflammation in mice [6]. Besides the important role of *P. gingivalis* in PDis, the gram-negative bacteria *Aggregatibacter actinomycetemcomitans* (*Aa*) has been implicated in the most aggressive forms of PDis. *Aa* was described as a potent immunoregulator of the periodontal host defense system and alveolar bone homeostasis [7]. Despite the known implication of *Aa* in the aggressive forms of PDis and the link clinical link between PDis and AD, which is already known to involve the participation of *P. gingivalis*, the possible implications of *Aa* in the etiology and/or progression of AD have not been reported to date.

Different serotypes of *Aa* are described based on the antigenicity of the O-polysaccharide (O-PS) component of its Lipopolysaccharide (LPS), with the a, b and c serotypes the most prevalent in humans [8–11]. In fact, the a, b and c serotypes are most prevalent in the adult population than the d, e and f serotypes [8]. The association of different *Aa* serotypes with periodontal health or disease depends on the variable structure and immunogenicity of their O-PS, where serotype a is associated with health, and serotypes b and c with disease [8]. In this sense, oral pathogenic bacteria may be associated with systemic inflammatory diseases as a consequence of bacteremia [12].

Several neurodegenerative diseases have been related to chronic inflammation, which affects microglial function [13]. Microglia are the phagocytic cells of the brain and it is now recognized that chronic activation of TLR2 and/or TLR4 on microglia leads to secretory responses and pro-inflammatory phenotype differentiation, known as the M1-phenotype [14]. In fact, the M1 pro-inflammatory phenotype is characterized by production of increased levels of IL-1β, IL-6, and TNF-α in response to chronic inflammatory attack; conversely, the M2 immuno-modulatory phenotype secretes increased levels of IL-4, IL-10, and transforming growth factor (TGF)-β1 in response to acute brain inflammation [14]. The increment in pro-inflammatory cytokine levels and microglial responses induces a chronic inflammatory encephalic environment that triggers the generation and accumulation of Aβ peptide [15], considered as having a central role in the pathogenesis of AD [16,17]. Interestingly, studies using TLR2/4-deficient mice revealed that the TLR2/4 pathway mediates the Aβ_1-42_ dependent pro-inflammatory cytokine release from innate immune cells [18]. Moreover, the M1-phenotype produces a burst of reactive oxygen and nitrogen species (ROS/RNS), and activates cytokine secretion in astrocytes, which together induce loss of neuronal function, contributing to the establishment of chronic brain inflammation [19,20]. Recently, it was reported that neuronal death during neurobruscellosis is a consequence of microglial TLR2-signaling induced by a lipoprotein of *Brucella abortus* and its inhibition induces failure in microglia activation and antigen elimination [21,22]. Based on these combined results, it seems reasonable to propose that activation of microglia towards the M1 phenotype is responsible for neuronal distress and death.

Although M2 cells can neutralize the presence of these inflammatory mediators, under constant and enhanced cytokine production M2 cells fail to achieve their homeostatic role, leading to M1 cell activation and consequent astrocyte induction [14,23]. These molecular and cellular processes generate a cellular response known as astrogliosis, which leads to oxidative stress, low import of glucose and oxygen, ROS and Aβ production, which affects neuronal physiology [16,23,24]. Thus, virulence factors of pathogenic bacteria are likely to play a role during the etiopathogenesis of neurodegenerative disorders, contributing to M1-phenotype polarization and astrogliosis.

In order to determine whether brain cells recognize the variability in LPS conformation of *Aa* serotypes, we treated rat cortical microglial and hippocampal mixed-cultures with different LPS serotypes of the bacteria *Aa* and quantified the generation of pro-inflammatory mediators and of Aβ_1-42_, and evaluated cell morphology and number.

## Methods

### Bacterial culture

In the present study, purified LPS of ATCC® 43717™ (serotype a), ATCC® 43718™(serotype b) or ATCC® 43719™ (serotype c) strains of *Aa* were used to treat cells as previously described [25]. *Aa* LPS was obtained as described previously [26]. Briefly, 1 × 10^10^ CFU/mL of *Aa* was resuspended in Tris-reagent and chloroform was added to incubate at room temperature for 15–30 min. Further, a supernatant was obtained by centrifugation for 10 min at 11.000 x*g* and the supernatant was purified by overnight lyophilization. Then, LPS was resuspended in 1 mL of MgCl_2_ 0.375 M in 95% ethanol and transferred to a 1.5 mL Eppendorf tube, and after complete mixing the suspension was centrifuged at 5.000 x*g* for 5 min. This step was repeated twice. The second supernatant was decanted and 1 mL of 100% EtOH was added and the suspension was centrifuged at 5.500 x*g* for 5 min. The final pellet was resuspended in 0.1 mL of endotoxin-free water. The LPS was visualized by sodium dodecyl sulphate-14% polyacrylamide gel electrophoresis (SDS-PAGE) and periodic acid-silver staining as previously described [27]. The figure depicting LPS of serotypes a, b or c of *Aa* was published previously as supplementary figure [28].”

### Mixed hippocampal cultures

The Ethics Committee for Animal Research of the Faculty of Dentistry, Universidad de Chile, approved the bioethical protocol used in this study (17085-ODO-UCH Protocol). All procedures were performed in accordance with the Guideline for the Care and Use of Laboratory Animals from the National Institutes of Health, USA. Animals were housed with food and water *ad libitum* and were euthanized under deep anesthesia to avoid animal suffering at each stage of the experiments. Cultures were prepared from 18-day-old embryos obtained from pregnant Sprague–Dawley rats as previously described [29]. Briefly, after removal brains were placed in a dish containing Hank’s-glucose solution and hippocampi were dissected by gentle mechanical dissociation in Hank’s-glucose solution. The cell suspension was centrifuged and resuspended in Dulbeco’s modified Eagle’s medium (DMEM) supplemented with 10% horse serum and plated on polylysine-treated plates. After 40 min, DMEM was replaced by Neurobasal medium supplemented with B-27. Cells were then maintained *in vitro* for 18–21 days at 37°C in a humidified 5% CO_2_ atmosphere prior to experimental manipulations.

### Microglial cultures

Rat cortical microglia were obtained as previously described [24]. Briefly, cerebral cortex from 1- to 3-day-old Sprague**–**Dawley either male or female rat pups were dissected in Hank’s-buffered salt solution and digested using 0.25% trypsin supplemented with 0.5 mg/mL DNase I. The mixed astrocyte/microglia cultures were incubated in DMEM containing 10% fetal bovine serum. After 13 days in culture, cells were vigorously stirred on a shaker at 2 x*g* for 72 h to detach microglial cells. Microglial cells were plated in DMEM supplemented medium until 80% of confluence.

### Treatments

To evaluate whether hippocampal cells recognize and respond differentially to the structural variability of LPS from different serotypes of *Aa*, mixed hippocampal cells or microglial cortical cells were treated with 50 ng/mL of *Aa* LPS for 48 h. Cells incubated with 50 ng/mL LPS of a or c serotypes of *Aa* were considered as controls; for negative controls (non induced, n.i.), cell cultures were supplemented with phosphate saline buffer (PBS) (1X), the LPS vehicle, as previously performed [25].

### Cytokine expression quantified by qPCR

Purification of the total cytoplasmic mRNA and synthesis of the first cDNA strand was performed as previously described [25,30]. The expression levels of the cytokines IL-1β, IL-4, IL-6, IL-10, IL-17 and TNF-α and receptors TLR2 and TLR4 mRNAs were analyzed by qPCR from 50 ng cDNA in a qPCR equipment (StepOnePlus®; Applied Biosystems, Singapore), using specific primers (Table 1) and a qPCR reagent (KAPA^TM^ SYBR® Fast qPCR; KAPA Biosystems, Woburn, MA, USA) following the manufacturer’s recommendations.

**Table 1.** Primers used in qPCR assays.

Target	*Forward primer*	*Reverse primer*
IL-1β	*5ʹtgtgatgaaagacggcacac3’*	*5ʹcttcttctttgggtctttgtttgg3’*
IL-4	*5ʹgtgatgcaggacacaaggtc3’*	*5ʹtcccggaaagtgaagatacg3’*
IL-6	*5ʹcccttcaggaacagctatgaa3’*	*5ʹacaacatcagtcccaagaagg3’*
IL-10	*5ʹagtggagcaggtgaagaatga3’*	*5ʹtcatggccttgtagacacctt3’*
TNF-α	*5ʹgcccagaccctcacactc3’*	*5ʹccactccagctgctcctct3’*
TLR2	*5ʹagatggccacaggactcaag3’*	*5ʹttgcagcatcctctgagattt3’*
TLR4	*5ʹccttgagaaagtggagaagtcc3’*	*5ʹgctaagaaggcgatacaattcg3’*
18S	*5ʹgggcccgaagcgtttacttt3’*	*5ʹttgcgccggtccaagaattt3’*

Table 1 shows the sequence of specific primers used to determine the expression levels of the cytokines IL-1β, IL-4, IL-6, IL-10, IL-17 and TNF-α and receptors TLR2 and TLR4 mRNAs.

### Cytokine secretion quantified by ELISA

The secretion levels of the cytokines IL-1β, IL-6, TNF-α (R&D Systems Inc., Minneapolis, MN, USA), and Aβ1-42 (ThermoFisher Scientific, Life Technologies, USA) were quantified from 50 to 100 μL cell culture supernatants by ELISA following the manufacturer’s instructions, evaluating the absorbance at 460 nm and 560 nm using an automated plate spectrophotometer (Synergy^TM^ HT, Bio-Tek Instrument Inc., Winooski, VT, USA) as previously described [25,30].

### Immunofluorescence analysis

To analyze the effects on cell morphology of the immune response induced by the different serotypes of *Aa* on cell cultures, we specifically stained neurons with the neuronal marker anti-microtuble-associated protein (MAP2), astrocytes with the specific marker anti-glial acidic protein, or microglia with the anti-ionized calcium binding adaptor molecule 1 (Iba1) and evaluated cell morphology by immunofluorescence. After LPS-stimulation, primary cultures were fixed in 4% paraformaldehyde, blocked and permeabilized for 2 h with 2.5% BSA and 0.3% Triton X-100 in PBS. Hippocampal cells were then incubated overnight at 4°C with the primary antibodies anti-MAP2 at 1:500 (Abcam®, Cambridge, MA, USA), or mouse monoclonal anti-GFAP (Cell Signaling Technologies (Danvers, MA, USA) at 1:300 or rabbit polyclonal anti-Iba1 (Abcam®, Cambridge, MA, USA) at 1:100 diluted in blocking solution, and washed three times with PBS. Microglial cells were only incubated overnight at 4°C with the primary antibody anti-Iba1, diluted in blocking solution at 1:300, and washed three times with PBS. Subsequently, cells were incubated with the secondary anti-goat antibody (Alexa Fluor® 633, Abcam®) at 1:2.000 or anti-rabbit antibody (Alexa Fluor® 488) and anti-mouse (Alexa Fluor® 635, Thermo Fisher Scientific) both at 1:400 diluted in blocking solution and added to cells according to the protocol previously described [31]. After 2 h incubation at room temperature, cells were washed 3 times with PBS and mounted in medium containing DAPI (Abcam®) in order to visualize the nuclei. Cells were examined on a Confocal C2 Plus Spectral Microscope (Nikon, USA) and images were processed using the Image J software (NIH, MD, USA). Images were obtained from at least five randomly chosen fields per coverslip, from at least three coverslips for each culture. The proportion of labeled cells was determined by counting cells positively stained by primary antibodies in relation to total cells stained with DAPI. Sholl analysis was performed using the Image J software. Three to five independent experiments were performed with cultures obtained from embryos from different rats.

### Cell viability assay

After stimulation with serotype b LPS for 48 h, cell viability was evaluated by the Live/Dead kit (Molecular Probes) as previously described [32]. Briefly, following manufacturer’s instructions, the culture medium was removed and cells were gently washed three times with warm PBS-glucose and incubated at room temperature for 20 min in the presence of 2 μM calcein AM ester and 1 μM ethidium homodimer in PBS-glucose. Live neurons were identified by green calcein fluorescence and dead neurons were identified by the red fluorescence of DNA-bound ethidium. Cells were examined and counted on a Nikon® Eclipse Ti-E at 20X magnification. At least three random fields were imaged per culture well (two replicate wells were used per experimental condition in each experiment). Three independent experiments were performed with different neuronal cultures.

### Data analysis

The qPCR data were presented as relative fold-change (2^−∆∆CT^ method) by normalizing the expression of cytokines or receptors to 18S rRNA. The ELISA data were calculated using a five-parameter logistic equation and were expressed as pg/mL. Data were statistically analyzed using SPSS 22.0 software (IBM Corp., Armonk, NY, USA). The normal distribution of the data was determined using the Shapiro-Wilk test. Differences among groups and multiple comparisons were analyzed by ANOVA-Tukey or Kruskal Wallis-Dunn tests for parametric or non-parametric distribution, respectively. Differences between two groups were analyzed by t-test. Differences were considered statistically significant at *p* < 0.05.

## Results

### Purified LPS of Aa triggers the expression of cytokines and toll-like receptors in microglial cells

**Figure 1.**
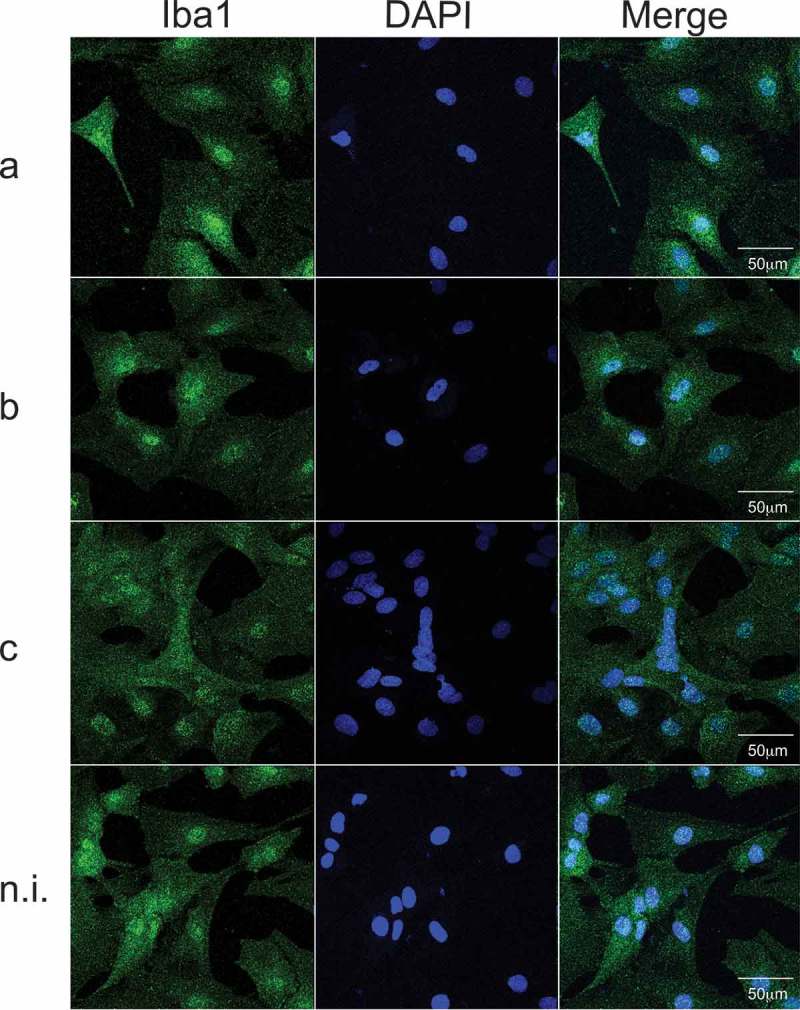
Cell morphology in microglial cultures, exposed to purified LPS (50 ng/mL) of serotypes a, b, or c of *Aa*; non-induced cultures were considered as control. In blue, the nuclei (DAPI), in green a microglia/macrophage-specific calcium-binding protein (Iba-1). The merged images show the colocalization of both stains. Scale bar: 50 µm.

Pro-inflammatory and immune-modulatory cytokine expression levels were quantified by qPCR on microglia treated with purified LPS (50 ng/mL) of serotype a (ATCC® 43717™), b (ATCC® 43718™) or c (ATCC® 43719™) of *Aa*. Figure 1 shows images of microglial cultures after treatment with LPS from different serotypes of *Aa*. An increment in the mRNA expression levels of IL-1β, IL-6, IL-17 and TNF-α was detected in serotype b-treated cells compared with the non-induced controls (Table 2) and to those treated with serotype a and c (not shown), respectively. When cells were exposed to serotype a, a significant increment in the expression levels of IL-4 and IL-10 was detected when compared to non-induced controls (Table 2) and to cells treated with serotype b or c (not shown). Higher expression levels of TLR2 mRNA was detected on microglia treated with LPS from serotype a or b compared with LPS from serotype non-induced controls (Table 2). Significant differences were also detected on TLR2 mRNA levels when cells exposed to b-serotype LPS were compared to cells exposed to LPS from serotypes a or c (not shown). Conversely, a higher expression level was detected for TLR4 mRNA when cells were exposed to all LPS serotypes, compared with non-induced controls (Table 2). A significant difference was also detected when the TLR4 mRNA levels of the cells treated with the LPS of serotype a, were compared with those that were treated with serotype c (not shown). No differences were detected when comparing TLR2 mRNA expression levels among microglia treated with serotype a, c or non-induced controls (not shown).

**Table 2.** Quantitative PCR analysis of cytokines profile and TLR2 and TLR4 mRNA expression in microglial cultures stimulated with purified LPS of serotypes a, b, or c of Aa.

IL-1β	Fold change	IL-4	Fold change	IL-6	Fold change	IL-10	Fold change
Serotype a	2.72 ± 2.21	Serotype a	9.96 ± 6.12	Serotype a	1.54 ± 1.88	Serotype a	4.29 ± 1.68
	p = 0.206		p < 0.0001		p = 0.693		p < 0.0001
Serotype b	7.75 ± 4.75	Serotype b	2.01 ± 1.81	Serotype b	4.61 ± 3.41	Serotype b	0.75 ± 0.35
	p < 0.0001		p = 0.772		p = 0.002		p = 0.685
Serotype c	1.89 ± 1.81	Serotype c	2.83 ± 2.13	Serotype c	0.76 ± 0.61	Serotype c	1.86 ± 0.96
	p = 0.526		p = 0.483		p = 0.985		p = 0.044
IL-17	Fold change	TNF-α	Fold change	TLR2	Fold change	TLR4	Fold change
Serotype a	1.51 ± 0.93	Serotype a	2.36 ± 2.57	Serotype a	10.39 ± 8.83	Serotype a	10.67 ± 5.28
	p = 0.069		p = 0.564	10.39 ± 8.83	p = 0.033		p < 0.0001
Serotype b	3.12 ± 1.51	Serotype b	8.87 ± 5.67	Serotype c	24.63 ± 11.83	Serotype b	7.21 ± 4.17
	p < 0.0001		p < 0.0001		p < 0.0001		p = 0.002
Serotype c	0.65 ± 0.21	Serotype c	2.23 ± 2.27	Non induced	5.95 ± 3.07	Serotype c	5.61 ± 3.84
	p = 0.786		p = 0.615	1,00 ± 0.00	p = 0.365		p = 0.02

### Mixed hippocampal cultures treated with purified LPS from serotype b of Aa express toll-like receptor 2, express and secrete pro-inflammatory cytokines, secrete Aβ1-42 and present altered neuronal morphology

**Figure 2.**
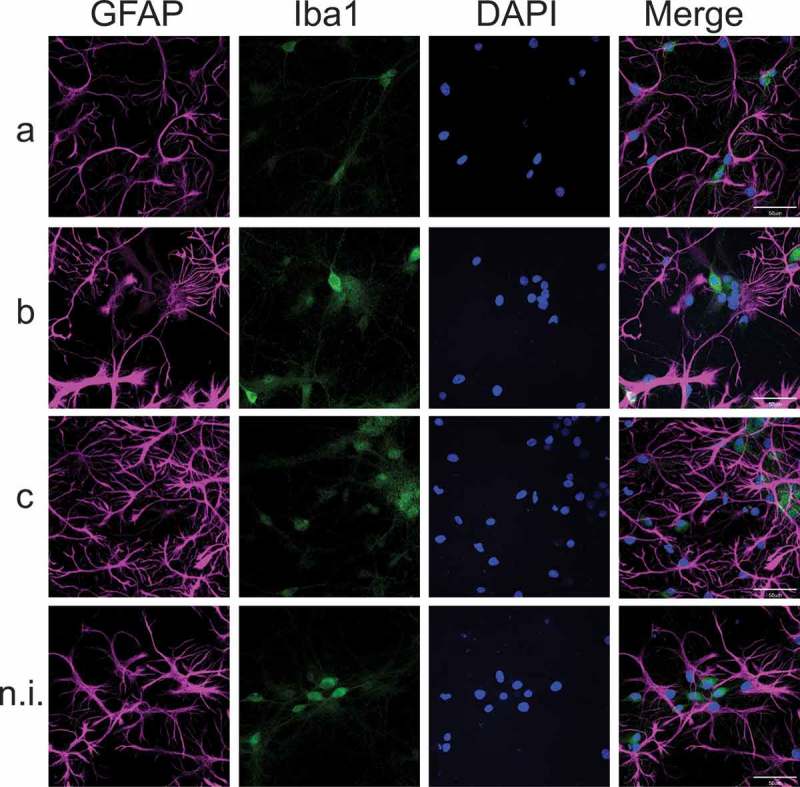
Cell morphology of mixed hippocampal cultures, exposed to purified LPS (50 ng/mL) of serotypes a, b, or c of *Aa*; non-induced cultures were considered as control. In blue, the nuclei (DAPI), in magenta, astrocytes stained with the glial acidic fibrillary protein (GFAP), in green, microglia stained with Iba-1. Merged images show the colocalization of the triple staining. Scale bar: 50 µm

In addition, the mRNA expression levels of the above cytokines were quantified on mixed hippocampal cultures stimulated with purified LPS from serotypes a, b, or c of *Aa*. The proportion of neurons, astrocytes and microglia present in mixed cultures was determined by immunoflurorescence (Supplementary Figure 1). After exposure to LPS from different serotypes of *Aa*, the immunostaining of the astrocytes and microglia present in these mixed hippocampal cultures did not reveal a significant difference neither in the number nor in the morphology of these cell types (Figure 2). However, as observed in pure microglial cultures (Table 2), an increment in the mRNA expression levels of IL-1β, IL-6, IL-17, TNF-α, TLR2 and TLR4 was detected in serotype b-treated cells compared to the non-induced controls (Table 3) and to those treated with serotype a or c (not shown). However, no differences were detected for TLR2 or TLR4 expression levels when cells were treated with c serotype of *Aa* when compared to non-induced controls (Table 3) or to cells treated with LPS from serotype a (not shown).

**Table 3.** Quantitative PCR analysis of cytokines profile and TLR2 and TLR4 mRNA expression in mixed hippocampal cultures stimulated with purified LPS of serotypes a, b, or c of Aa.

IL-1β	Fold change	IL-4	Fold change	IL-6	Fold change	IL-10	Fold change
Serotype a	3.82 ± 3.61	Serotype a	4.01 ± 2.37	Serotype a	2.07 ± 1.45	Serotype a	6.17 ± 3.30
	p = 0.942		p = 0.015		p = 0.419		p = 0.02
Serotype b	24.81 ± 16.44	Serotype b	1.37 ± 2.20	Serotype b	5.82 ± 2.76	Serotype b	3.28 ± 1.78
	p = 0.003		p = 0.788		p = 0.001		p = 0.075
Serotype c	4.21 ± 4.58	Serotype c	1.45 ± 1.37	Serotype c	2.23 ± 1.42	Serotype c	2.18 ± 1.47
	p = 0.922		p = 0.746		p = 0.323		p = 0.454
IL-17	Fold change	TNF-α	Fold change	TLR2	Fold change	TLR4	Fold change
Serotype a	1.16 ± 1.04	Serotype a	2.93 ± 1.56	Serotype a	3.85 ± 1.08	Serotype a	2.31 ± 1.33
	p = 0.802		p = 0.052	10.39 ± 8.83	p = 0.588		p = 0.033
Serotype b	6.00 ± 3.70	Serotype b	6.25 ± 3.29	Serotype c	9.98 ± 5.86	Serotype b	3.46 ± 1.48
	p = 0.001		p < 0.0001		p = 0.005		p = 0.002
Serotype c	1.58 ± 0.97	Serotype c	2.31 ± 1.56	Non induced	1.49 ± 0.99	Serotype c	1.83 ± 1.11
	p = 0.612		p = 0.277	1,00 ± 0.00	p = 0.990		p = 0.116

Table 3 shows the quantitative PCR analysis of IL-1β, IL-4, IL-6, IL-10, IL-17, TNF-α and TLR2 and TLR4 mRNA expression in microglia cell cultures treated with purified LPS (50 ng/mL) of serotypes a, b, or c of *Aa*. For relative expression, cytokine mRNA expression in non-induced microglia was used as control. Data are presented as fold-change and shown as mean ± SE for 10 independent experiments. Each experiment was performed in duplicate. p values: in comparison to non-induced controls. IL: Interleukin, TLR: Toll-like receptor, TNF: Tumor Necrosis Factor.

The secretion levels of the pro-inflammatory cytokines IL-1β, IL-6, and TNF-α were also quantified by ELISA on primary mixed hippocampal cultures exposed to purified LPS from serotypes a, b, or c of *Aa* (Table 4). An increment in the secretion levels of IL-6 and TNF-α were detected in cultures treated with LPS from serotype b, compared with non-induced controls (Table 4) and to cultures treated with LPS from serotypes a or c (not shown). Also, the secretion levels of IL-1β were higher in cells exposed to LPS from serotype b when compared to cells treated with LPS from serotype a (not shown).

**Table 4.** Cytokines and Aβ 1–42 secretion profile in mixed hippocampal cultures stimulated with purified LPS of serotypes a, b, or c of Aa.

IL-1β	Fold change	IL-6	Fold change	TNF-α	Fold change	Aβ_1-42_	Fold change
Serotype a	0.903	Serotype a	1,009	Serotype a	0.613	Serotype a	1,545
	p = 0.978		p = 1.000		p = 0.585		p = 0.035
Serotype b	1,624	Serotype b	1,409	Serotype b	1,772	Serotype b	2,001
	p = 0.023		p = 0.020		p = 0.038		p < 0.0001
Serotype c	1,216	Serotype c	1,215	Serotype c	1,134	Serotype c	1,831
	p = 0.635		p = 0.274		p = 0.955		p < 0.0001

Table 4: The Table shows cytokine and Aβ_1-42_ secretion levels in mixed hippocampal cultures treated with purified LPS (50 ng/mL) of serotypes a, b, or c of *Aa*. Analysis of secretion levels of the cytokines IL-1β, IL-6, and TNF-α and of Aβ_1-42_ in mixed hippocampal cultures treated with purified LPS (50 ng/mL) of serotypes a, b, or c of *Aa* was performed. Data are presented as fold of control calculated from 10 independent experiments. p values: in comparison to non-induced controls. The concentrations of each molecule in the cell culture supernatants of non-induced controls were in pg/mL (IL1β: 143.54 ± 21.47; IL-6: 174.67 ± 29.49; TNF-α: 79.88 ± 26.30 and Aβ_1-42_: 24.81 ± 9.03, presented as mean ± SD). IL: Interleukin, TNF: Tumor Necrosis Factor.

**Figure 3.**
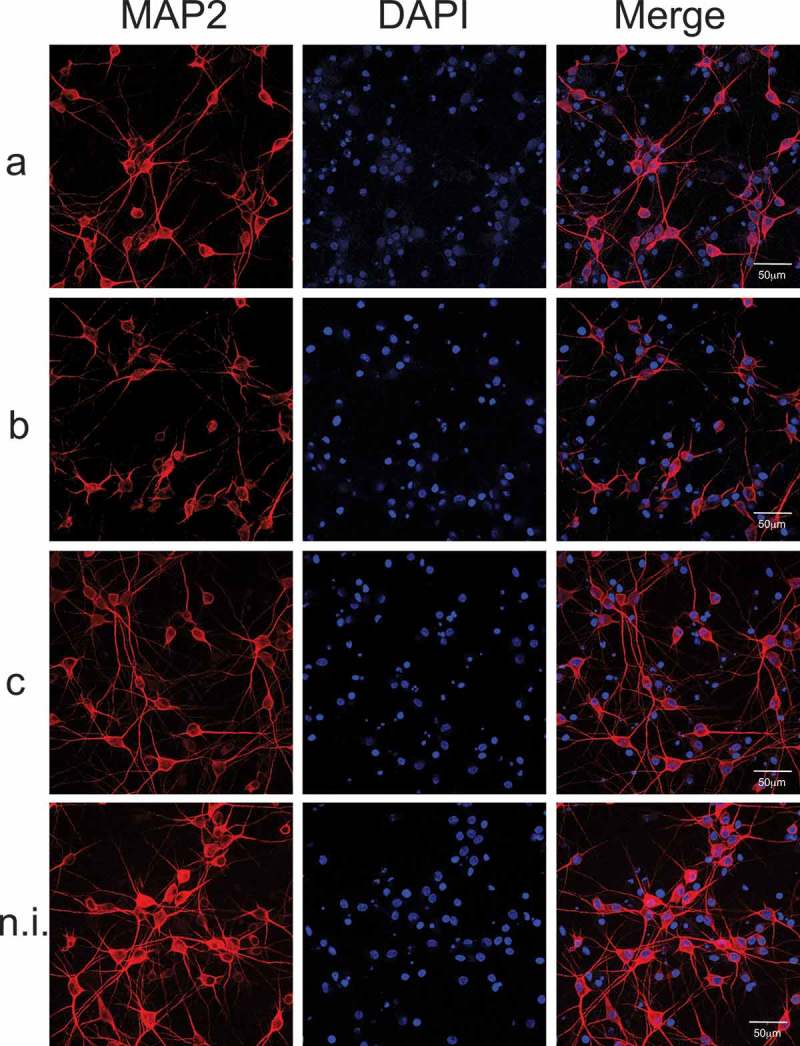
Neuronal morphology of mixed hippocampal cultures. (A) Neuronal morphology was evaluated by MAP2 staining. Mixed hippocampal cultures were exposed to purified LPS (50 ng/mL) of serotypes a, b, or c of *Aa*; non-induced cultures were considered as control. In blue, the nuclei (DAPI), in red the microtubule-associated protein specific of neurons (MAP2), the merged images show the colocalization of both stains. Scale bar: 50 µm.

Importantly, the levels of Aβ1-42 showed a slight but significant increase on the supernatants obtained from primary mixed hippocampal cultures treated with LPS from different serotypes of *Aa*, with a statistically significant effect in cells exposed to serotype b LPS (Table 4), as detected by ELISA. To investigate the effects of the different patterns of expression of pro-inflammatory cytokines induced by exposure to the different serotypes of *Aa* on the morphology of the neurons, we employed immunofluorescence techniques to specifically stain neurons in primary mixed hippocampal cultures. To this aim, we used an antibody against a neuron-specific microtubule-associated protein (MAP), as previously described [31]. MAP2 isoforms are expressed only in neuronal cells, specifically in the perikarya and in dendrites, where they are enriched. Cells were treated with purified LPS of serotypes a, b, or c of *Aa* (50 ng/mL) and immunolabeled with antibody anti-MAP2, plus DAPI stain to identify the nuclei of all cells (Figure 3).

When primary mixed hippocampal cultures were exposed to purified LPS of *Aa*, no differences were detected in the proportion MAP2/DAPI (neuron/nuclei) staining among the different experimental conditions (Supplementary Figure 2). Also, nuclei staining counting revealed no significant difference in the total number of cells after LPS from *Aa* challenge among the groups (not shown), indicating that LPS-treatments did not induce hippocampal cell loss, at least in the experimental conditions employed.

**Figure 4.**
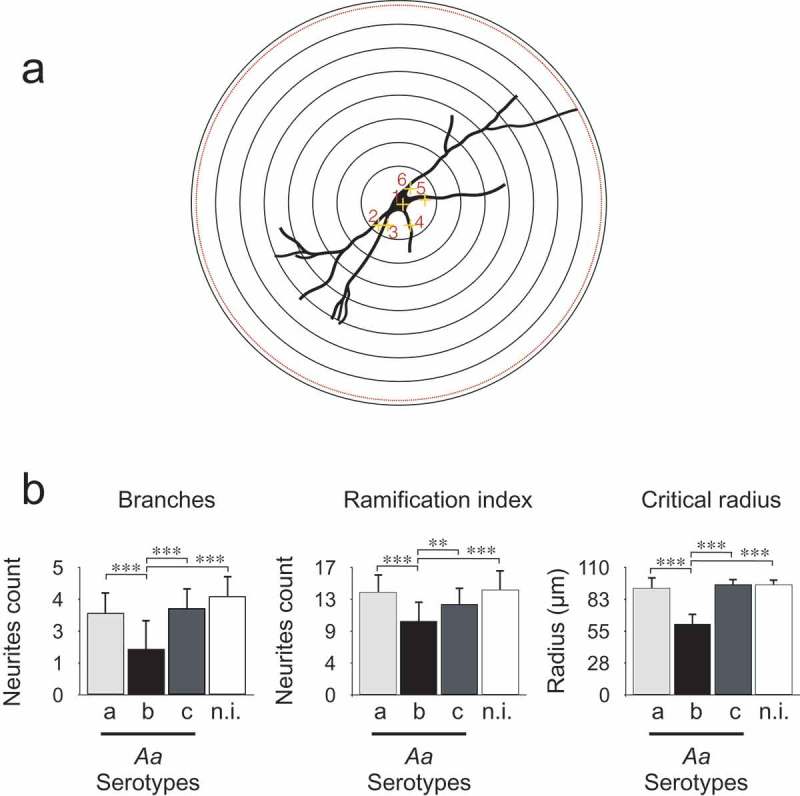
Quantification of dendritic arborization by Sholl analysis of neurons in mixed hippocampal cultures. (A) Sholl analysis scheme. The Sholl technique describes neuronal arbors from the initial marks. Each neuron is marked in the nuclei and the neurites, and a program creates concentric shells around nuclei from 10 to 100 microns and count how many times the neurites intersect each shell. The program quantifies the number of branches or initial neurites, the Schoenen ramification index (the ratio of maximum intersection and the primary number of neurites) and the critical radius (represents at which distance is the longest neurite). (B) Sholl analysis of hippocampal neurons treated with LPS from different serotypes of *Aa* and stained with MAP2. The Sholl analysis was performed on mixed hippocampal cultures treated with 50 ng/mL of purified LPS of serotypes a, b, or c of *Aa* to quantify number of branches, ramification index and critical radius. Data are presented as the mean ± SD for 5 independent experiments. ***p < 0.001.

Nevertheless, dramatic morphological changes characterized by dendrites shrinking and disappearance were detected in neurons treated with serotype b-purified LPS of *Aa*, compared with serotypes a, c, or non-induced controls, as shown in Figure 3. To confirm that the dramatic effect of serotype b LPS on neuronal morphology did not affect cell viability, we performed the live dead assay, which allows to identify live cells with calcein-AM and dead cells with ethidium homodimer. No significant changes were observed in cell viability when mixed hippocampal cultures were treated with LPS from serotype b (Supplementary Figure 3). However, neurons treated with serotype b of *Aa* exhibited significantly lower values for the number of branches, the ramification index (arborization) and the critical radius, when compared with those of cells exposed to serotype a, c or with non-induced controls, as quantified by the Sholl analysis (Figure 4).

## Discussion

Bacterial LPS is a potent virulence factor that induces a strong immune response in host cells. The detection of LPS in brain samples was associated with a cerebral inflammatory response, characterized by activated microglia determining pro-inflammatory and pro-amyloidogenic reactions in brain cells [13,33]. A recent report showed that under physiological conditions, LPS enters the rat brain by a lipoprotein-mediated transport mechanism [34]. Thus, the chronic presence of LPS of periodontopathogenic bacteria associated to PDis could represent a risk factor for the function of brain cells. Indeed, recent studies have evaluated the effect of the highly prevalent periodontopathogenic bacteria *P. gingivalis* on neuroinflammation, showing that this bacterial infection induces the secretion of inflammatory cytokines and causes cognitive defects [35,36]. Also, oral infection with *P. gingivalis* aggravates AD features in an AD transgenic rat model [6] and promotes brain inflammation, neurodegeneration and amyloid beta production in wild type mice [37]. Interestingly, capsular polysaccharide antigens from *P. gingivalis* have different immunogenicity potentials that determine their pathogenicity and immunogenicity, in analogy to what occurs with the LPS from different serotypes of *Aa* [25,38].

Our results show that the LPS from serotype b was remarkably more immunogenic compared to the LPS from the other serotypes, inducing pro-inflammatory cytokine production either in microglia or in mixed hippocampal cells, as it does in other immune cells of the host, as we previously described [25,39]. These findings evidence a discriminatory capacity of the brain immune response for this periodontopathogen. Of note, experimental infections with *P. gingivalis* to evaluate neuroinflammation and cognitive impairment have not considered the variability of the different antigens from each bacterial serotype of *P. gingivalis* [35,36]. Nevertheless, our results show that the serotype b of *Aa* directly impacts on the pattern of cytokine secretion and, consequently, on neuronal morphology. Interestingly, it has been shown that *Aa* expresses curli proteins that form amyloid fibers that bind thioflavin T and exhibit yellow-green birefringence under polarized light [40]. Curli proteins are involved in adherence to and invasion of host cells, cell aggregation, and biofilm formation [41]. It is possible to surmise that the immunological mechanisms activated in response to fimbrial curli *Aa* protein are similar to those involved in the response to Aβ amyloidogenic aggregates.

Our results demonstrate that LPS of serotype b induces the expression and secretion of pro-inflammatory cytokines and increases the expression of TLR2 and TLR4 both in microglia and mixed hippocampal cultures; in contrast, TLR4 expression increased in response to all stimuli only in microglial cultures. Of note, according to our previously published results, the LPS variability was recognized by TLR4, but the inflammatory response was TLR2 dependent [39]. In this context, the response detected in microglia could be associated with the recognition of the LPS from different serotypes of *Aa* via TLR4. Moreover, the pro-inflammatory signaling observed in both types of cell cultures might be related to the increased expression of TLR2.

In the case of *Aa*, the LPS from serotype b specifically induced release of inflammatory cytokines by microglia and in mixed hippocampal cultures, but only in the last case an important change in neuronal morphology was observed, with a clear and strikingly neuritic beading (Figures 3 and 4). Interestingly, neuritic beading induced by activated microglia is an early feature of neuronal dysfunction, which occurs in response to Aβ-induced inflammatory responses involving astrocytes and microglia [24,42]. In addition, neuroinflammation induced by LPS enhances Aβ generation [43]. Accordingly, we found that mixed hippocampal cultures treated with LPS from *Aa* presented an increase in Aβ_1-42_ production (Table 4). Based on these results, we propose that a feed forward injurious cycle would emerge, in which the increase in the levels of pro-inflammatory cytokines would activate amyloidogenic secretases to produce increased Aβ_1-42_ levels [44], and the increase in the levels of Aβ_1-42_ would activate glial cells to produce pro-inflammatory cytokines [24].

Chronic infection of the periodontium together with the continuous up-regulation of pro-inflammatory responses and immune mediators may contribute to systemic sequels including diabetes and cardiovascular diseases [45]. Of note, it has been known for a long time that some cytokines can directly and rapidly cross the blood brain barrier and reach the brain [46]. The presence of chronic inflammatory mediators in the brain can induce M1 differentiation in the absence of antigens [47]. The subsequent presence of LPS from a highly immunogenic keystone bacterium would thus exacerbate the inflammatory phenomenon, whereas if the LPS is derived from a pathobiont it may counteract inflammation [48].

In conclusion, we report here that serotype b of the periodontopathogen *Aa*, associated with severe forms of PDis, caused specific inflammatory and immune responses in brain cells. These combined responses could increase the risk of AD, considering that a strong relationship between neuroinflammation and cognitive decline has been demonstrated recently [49]. Our results involve for the first time another important microbial agent in the etiology of the aggressive forms of PDis, the *Aa*, in the molecular mechanisms of AD pathogenesis. Thus, our findings indicate that *Aa* might be part of the consortium of periodontal bacteria playing a role in the development of AD. It is important to emphasize, however, that this is an in vitro study. Future in vivo studies are needed to verify if the Aa bacterium is actually able to enter the brain, increasing the relevance of oral infection by this microorganism in the context of AD.
