# Supplementary figures and images for: Serotype b of Aggregatibacter actinomycetemcomitans triggers pro-inflammatory responses and amyloid beta secretion in hippocampal cells: a novel link between periodontitis and Alzheimer´s disease?
Source: J Oral Microbiol. 2019 Apr 15;11(1):1586423. doi: 10.1080/20002297.2019.1586423 (PMC6484476; doi:10.1080/20002297.2019.1586423)

# Supplementary Figure 1

A

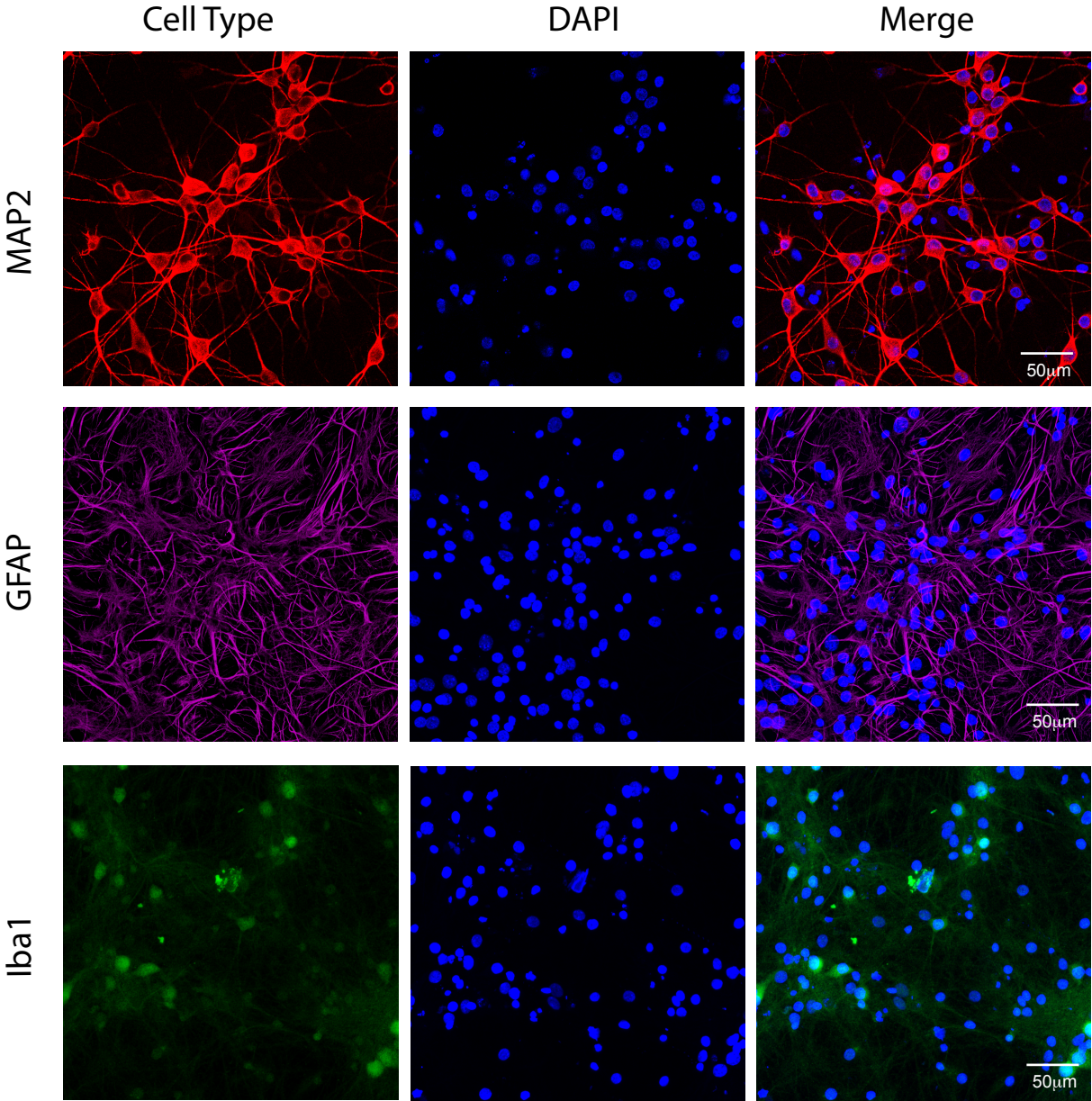

B

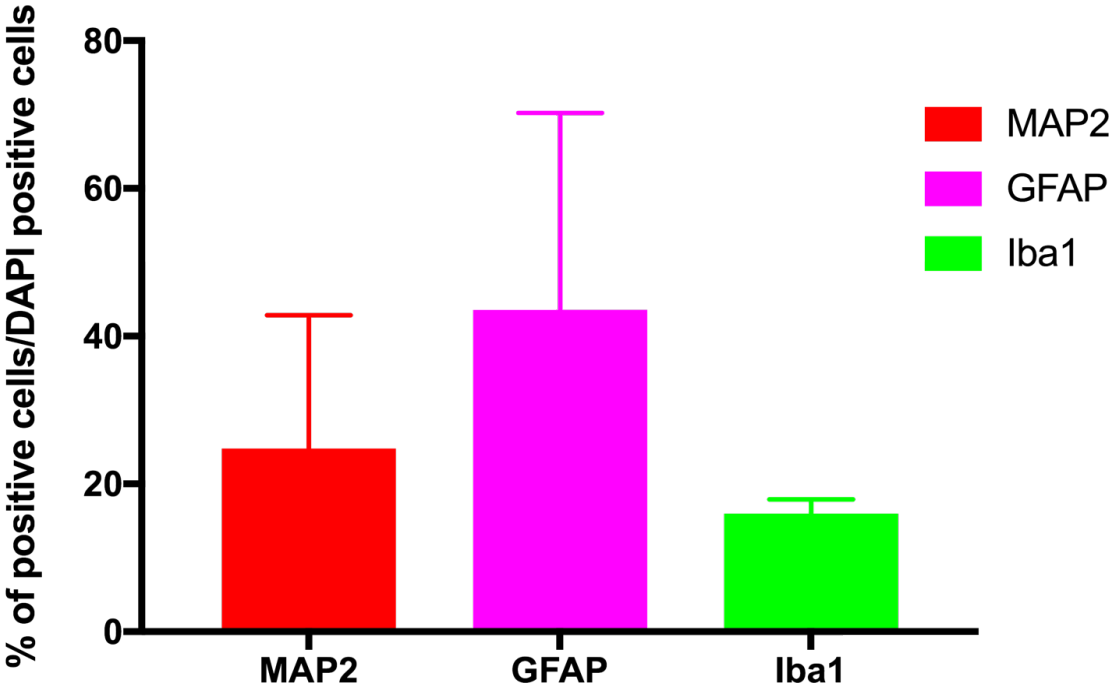

Supplement: Supplemental Material [file ZJOM_A_1586423_SM4576.zip › supplemental data/Supplementary Figure 1_.pdf]

# Supplementary Figure 2

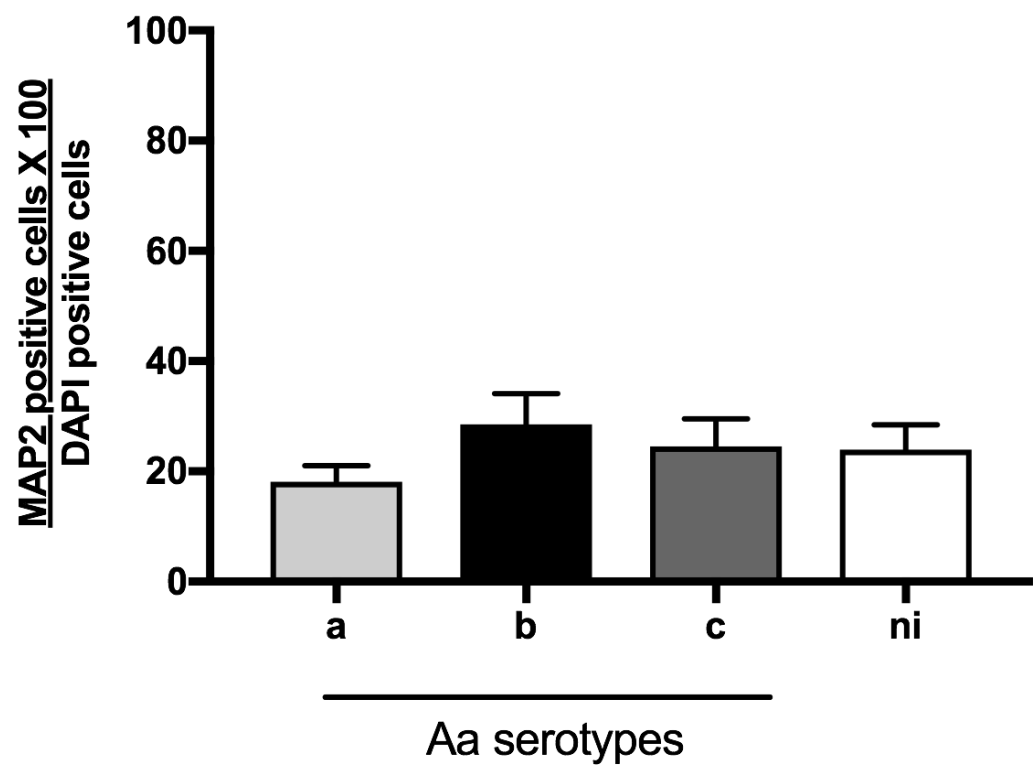

Supplement: Supplemental Material [file ZJOM_A_1586423_SM4576.zip › supplemental data/Supplementary Figure 2_.pdf]

# Supplementary Figure 3

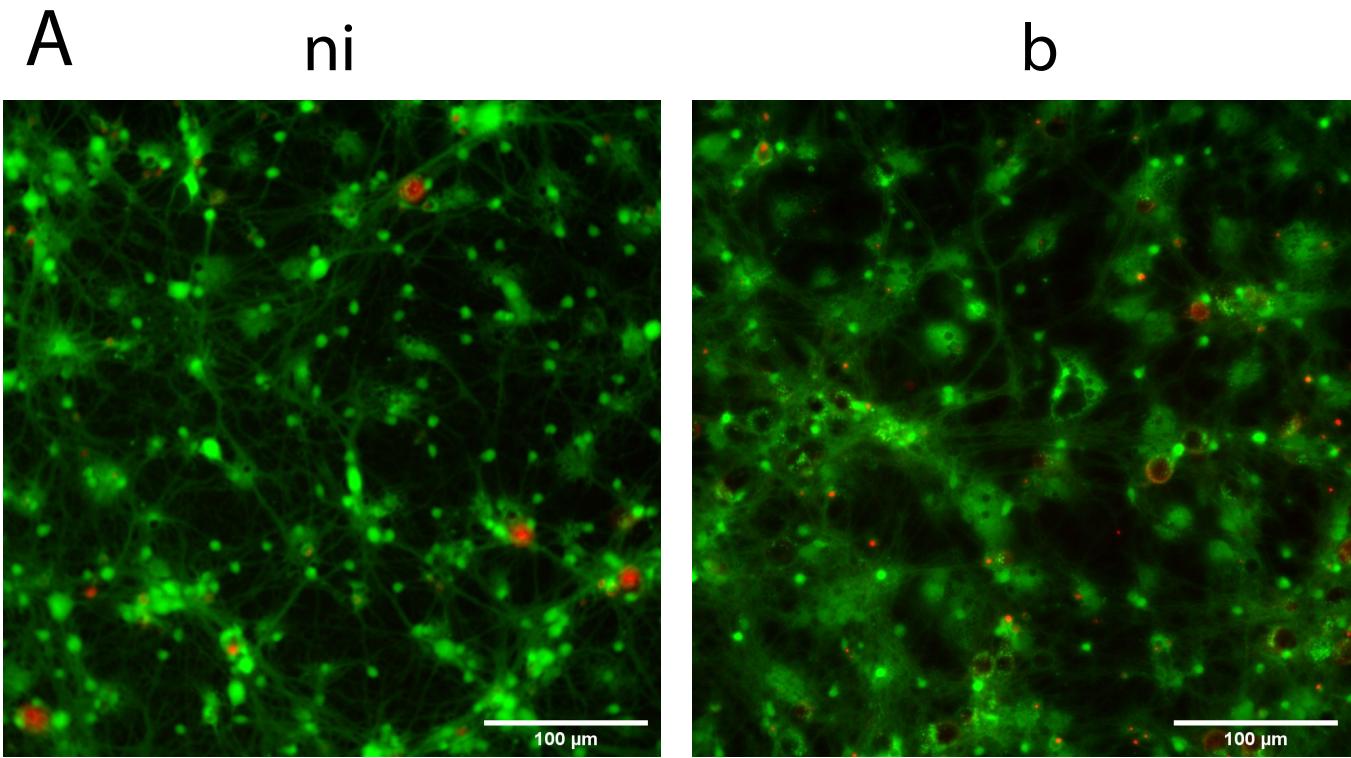

B

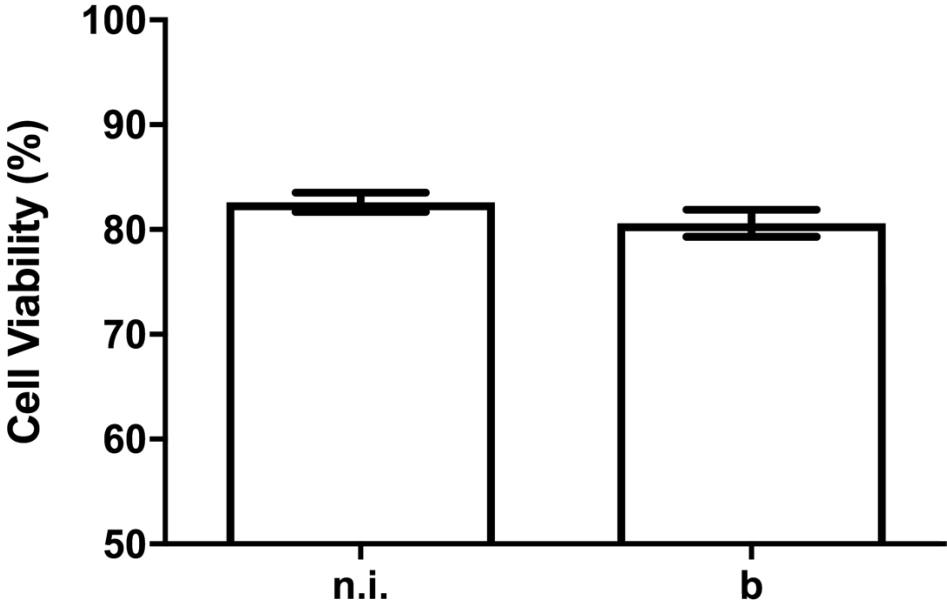

Supplement: Supplemental Material [file ZJOM_A_1586423_SM4576.zip › supplemental data/Supplementary Figure 3_.pdf]
